# Supplementary material for: Curvature sensing lipid dynamics in a mitochondrial inner membrane model
Source: Commun Biol. 2024 Jan 5;7:29. doi: 10.1038/s42003-023-05657-6 (PMC10770132; doi:10.1038/s42003-023-05657-6)
Supplement: Supplementary file 2 — Supporting Information [file 42003_2023_5657_MOESM2_ESM.pdf]

Supporting Information: Curvature Sensing Lipid Dynamics in a Mitochondrial Inner Membrane Model

Vinaya Kumar Golla,[1,2] Kevin J. Boyd,[1,3] and Eric R. May*,[1]

[1] *Department of Molecular and Cell Biology, University of Connecticut, Storrs, CT 06269, United States of America*

[2] *Present address: Department of Cell Biology,University of Virginia School of Medicine, Charlottesville, VA 22903, United States of America*

[3] *Present address: NVIDIA, 2860 County Hwy G4, Santa Clara, CA 95051, United States of America*

* E-mail: eric.may@uconn.edu

Supporting Information Available

The supporting information contains Tables S1-S3 and Figures S1-S24.

Table S1: Lipid composition of simulated membrane systems

Label	Systems	Geometry	r_{cyl} (nm)	%POPC	%POPE	%DOPE	% CDL^{-1}	CDL^{-2}	# CG Particles
A	POPC/Test(POPC) (4:1)	IMM	10	100	0	0	0	0	1,571,829
B	POPC/POPE (4:1)	IMM	10	80	20	0	0	0	1,569,060
C	POPC/DOPE (4:1)	IMM	10	80	0	20	0	0	1,566,422
D	POPC/CDL^{-2} (4:1)	IMM	10	80	0	0	0	20	1,591,442
E	POPC/POPE/CDL^{-2} (3:1:1)	IMM	10	60	20	0	0	20	1,597,792
F	POPC/DOPE/CDL^{-2} (3:1:1)	IMM	10	60	0	20	0	20	1,589,209
G	POPC/POPE/CDL^{-1} (3:1:1)	IMM	10	60	20	0	20	0	1,592,212
H	POPC/DOPE (4:1)	Flat	n/a	80	0	20	0	0	7634
I	POPC/POPE/CDL^{-2} (3:1:1)	IMM	15	60	20	0	0	20	1,601,473
J	POPC/POPE/CDL^{-2} (3:1:1)	IMM	5	60	20	0	0	20	1,590,689

Table S2: Estimated mean ($\times$ 10^{-2} nm^{-1}) and Gaussian (Gauss) ($\times$ 10^{-3} nm^{-2}) curvatures. To exclude outliers, the average mean and gaussian curvatures are calculated based on the values lying within the range of -0.04 to 0.04 and -0.004 to 0.004, respectively. Averages were computed over the final 500 ns of 4 μs simulations, where frames were collected every 25 ns. All systems have a 10 nm cylinder radius (r_{cyl}), unless otherwise noted.

System	Layer	Junction		Cylinder		Flat	
		Mean	Gauss	Mean	Gauss	Mean	Gauss
POPC/Test(POPC)	Outer	-3.03 ± 0.04	-6.6 ± 0.0	4.12 ± 0.01	-0.2 ± 0.0	-1.48 ± 0.01	0.0 ± 0.0
	Inner	0.81 ± 0.02	-5.0 ± 0.0	-6.08 ± 0.02	0.2 ± 0.0	0.93 ± 0.01	0.3 ± 0.0
POPC/POPE	Outer	-3.23 ± 0.02	-6.8 ± 0.0	4.14 ± 0.01	-0.2 ± 0.0	-1.39 ± 0.01	0.0 ± 0.0
	Inner	0.62 ± 0.02	-4.9 ± 0.0	-6.05 ± 0.02	0.0 ± 0.0	0.96 ± 0.01	0.3 ± 0.1
POPC/DOPE	Outer	-3.16 ± 0.02	-6.7 ± 0.0	4.11 ± 0.01	-0.3 ± 0.0	-1.39 ± 0.01	0.0 ± 0.0
	Inner	0.69 ± 0.02	-4.9 ± 0.0	-6.00 ± 0.03	0.0 ± 0.0	0.96 ± 0.01	0.3 ± 0.0
POPC/CDL^{-2}	Outer	-3.11 ± 0.03	-6.4 ± 0.0	4.23 ± 0.01	-0.1 ± 0.0	-1.51 ± 0.01	0.0 ± 0.0
	Inner	0.81 ± 0.01	-5.1 ± 0.0	-6.31 ± 0.02	0.3 ± 0.0	0.85 ± 0.01	0.2 ± 0.0
POPC/POPE/CDL^{-2}	Outer	-3.28 ± 0.03	-6.4 ± 0.0	4.31 ± 0.01	0.0 ± 0.0	-1.52 ± 0.01	0.0 ± 0.0
	Inner	0.57 ± 0.02	-5.2 ± 0.0	-6.40 ± 0.01	0.4 ± 0.0	0.93 ± 0.02	0.2 ± 0.0
POPC/DOPE/CDL^{-2}	Outer	-3.12 ± 0.02	-6.3 ± 0.0	4.24 ± 0.09	-0.1 ± 0.0	-1.54 ± 0.02	0.0 ± 0.0
	Inner	0.63 ± 0.01	-4.4 ± 0.1	-6.24 ± 0.02	0.3 ± 0.0	1.01 ± 0.03	0.2 ± 0.0
POPC/POPE/CDL^{-1}	Outer	-3.09 ± 0.02	-6.5 ± 0.0	4.36 ± 0.02	0.0 ± 0.0	-1.55 ± 0.01	0.0 ± 0.0
	Inner	0.74 ± 0.03	-4.9 ± 0.0	-6.40 ± 0.02	0.6 ± 0.1	0.94 ± 0.01	0.3 ± 0.1
POPC/POPE/CDL^{-2} (r_{cyl} = 15 nm)	Outer	-4.20 ± 0.04	-4.5 ± 0.0	3.04 ± 0.01	0.0 ± 0.0	-2.51 ± 0.02	0.1 ± 0.0
	Inner	1.71 ± 0.01	-3.6 ± 0.0	-4.10 ± 0.01	0.2 ± 0.0	1.48 ± 0.01	0.2 ± 0.0
POPC/POPE/CDL^{-2} (r_{cyl} = 5 nm)	Outer	-1.00 ± 0.04	-10.4 ± 0.1	7.43 ± 0.02	0.2 ± 0.0	-0.95 ± 0.01	0.0 ± 0.0
	Inner	-1.50 ± 0.07	-9.7 ± 0.4	-10.73 ± 0.02	-0.8 ± 0.4	0.43 ± 0.01	-0.9 ± 0.1

Table S3: The estimated lipid enhancement and depletion factors based on eq.1 for final 1 μs. The errors were estimated using the block averaging method with a block size of 80 ns. All systems have a 10 nm cylinder radius (r_{cyl}), unless otherwise noted.

System	Layer	Lipid type	Enrichment or depletion factor		
			Junction	Cylinder	Flat
POPC/Test(POPC)	Outer	Test	0.1 ± 0.1	-0.2 ± 0.09	0.1 ± 0.05
	Inner		-0.6 ± 0.08	-0.7 ± 0.07	0.6 ± 0.04
POPC/POPE	Outer	POPE	2.7 ± 0.08	-2.1 ± 0.07	-0.9 ± 0.05
	Inner		-1.8 ± 0.05	2.5 ± 0.07	0.2 ± 0.04
POPC/DOPE	Outer	DOPE	2.9 ± 0.07	-0.9 ± 0.07	-1.2 ± 0.03
	Inner		-0.8 ± 0.09	2.5 ± 0.12	-0.8 ± 0.05
POPC/CDL^{-2}	Outer	CDL^{-2}	3.0 ± 0.07	-3.4 ± 0.06	0.1 ± 0.03
	Inner		-1.7 ± 0.06	3.3 ± 0.06	0.2 ± 0.04
POPC/POPE/CDL^{-2}	Outer	POPE	1.5 ± 0.07	-1.8 ± 0.03	-0.1 ± 0.05
		CDL^{-2}	2.7 ± 0.06	-3.0 ± 0.04	-0.2 ± 0.03
	Inner	POPE	-1.5 ± 0.08	1.1 ± 0.1	0.5 ± 0.03
		CDL^{-2}	-2.4 ± 0.1	3.0 ± 0.03	0.6 ± 0.07
POPC/DOPE/CDL^{-2}	Outer	DOPE	2.0 ± 0.07	-1.1 ± 0.07	-0.7 ± 0.04
		CDL^{-2}	2.1 ± 0.05	-0.9 ± 0.06	-0.8 ± 0.04
	Inner	DOPE	-1.4 ± 0.09	0.7 ± 0.05	0.8 ± 0.06
		CDL^{-2}	-0.1 ± 0.07	2.5 ± 0.08	-0.8 ± 0.03
POPC/POPE/CDL^{-1}	Outer	POPE	1.9 ± 0.09	-1.9 ± 0.08	-0.1 ± 0.03
		CDL^{-1}	4.3 ± 0.12	-3.5 ± 0.07	-0.7 ± 0.04
	Inner	POPE	-0.7 ± 0.07	1.9 ± 0.04	-0.2 ± 0.04
		CDL^{-1}	-0.7 ± 0.07	3.1 ± 0.05	-0.7 ± 0.06
POPC/POPE/CDL^{-2} (r_{cyl}= 15 nm)	Outer	POPE	2.1 ± 0.08	-1.3 ± 0.07	-1.6 ± 0.08
		CDL^{-2}	3.1 ± 0.06	-2.5 ± 0.03	-1.3 ± 0.07
	Inner	POPE	-0.5 ± 0.07	1.1 ± 0.03	-0.2 ± 0.13
		CDL^{-2}	-1.1 ± 0.03	2.1 ± 0.05	-0.4 ± 0.07
POPC/POPE/CDL^{-2} (r_{cyl}= 5 nm)	Outer	POPE	1.8 ± 0.06	-1.6 ± 0.08	-0.2 ± 0.03
		CDL^{-2}	3.2 ± 0.05	-4.7 ± 0.03	0.1 ± 0.01
	Inner	POPE	0.2 ± 0.05	1.2 ± 0.06	-0.2 ± 0.03
		CDL^{-2}	-1.1 ± 0.05	4.5 ± 0.07	-0.3 ± 0.02

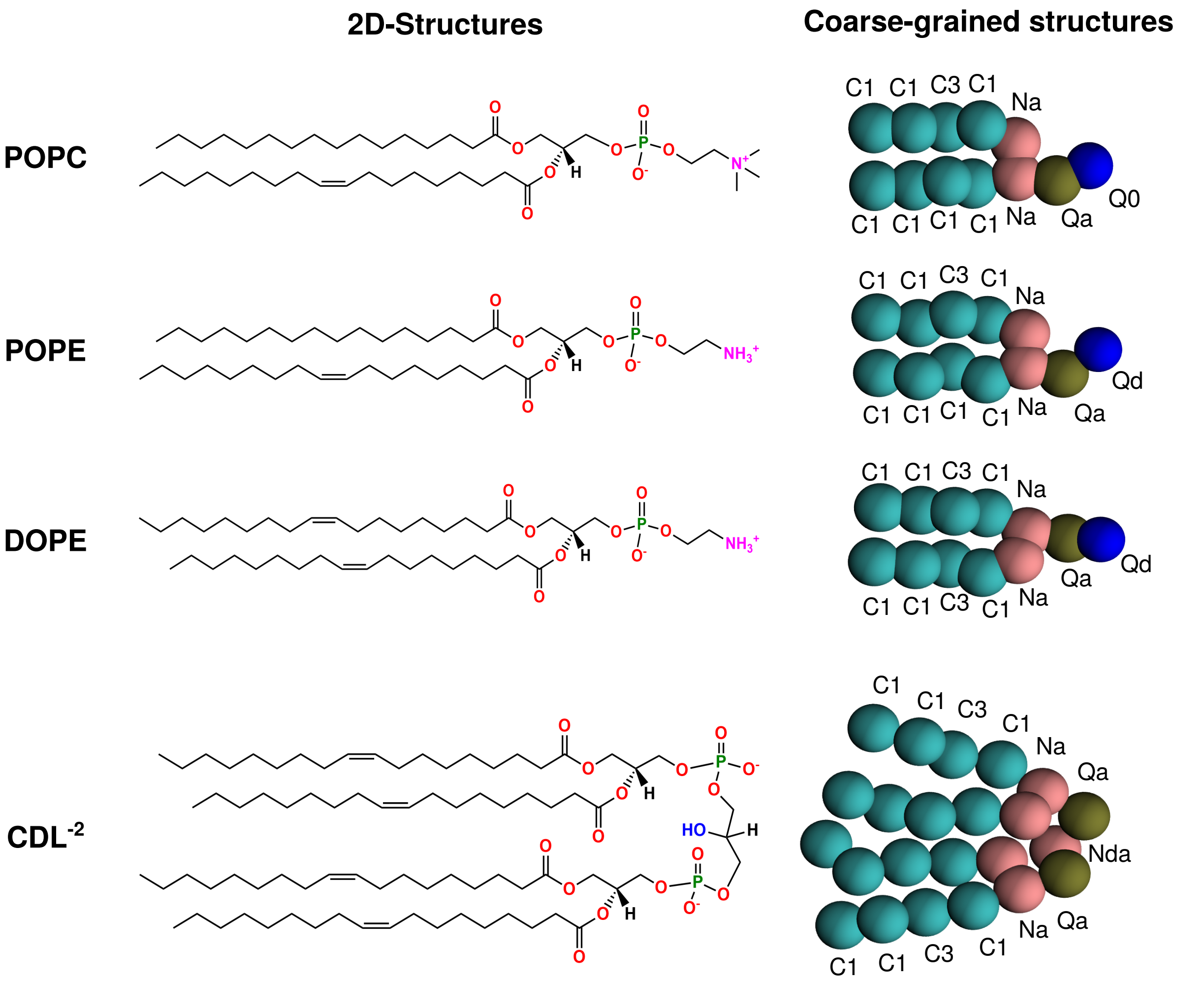

Figure S1: The 2D chemical structures of the different lipids used in the simulation study (except CDL^{-1} is not shown) are shown in the left column. In the right column, the corresponding CG representations and MARTINI bead types are shown.

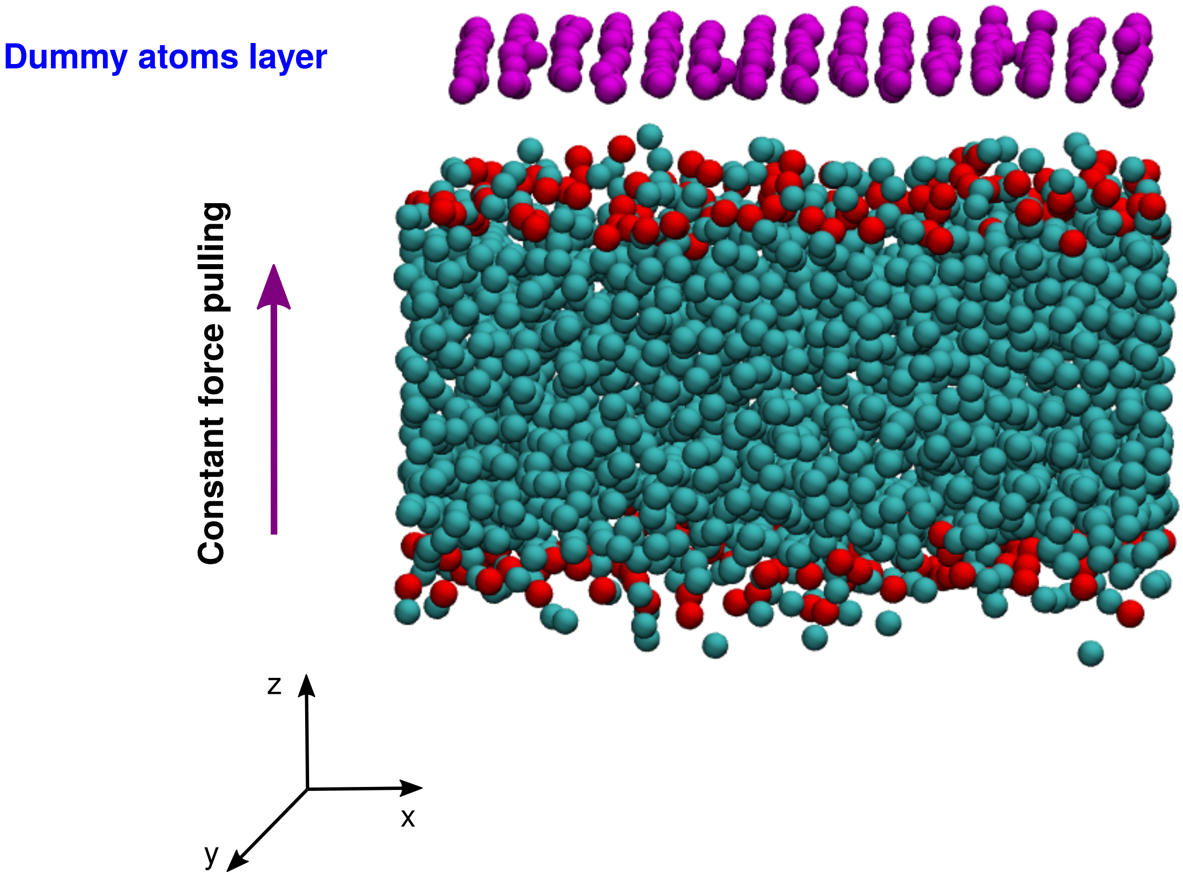

Figure S2: Representation of the flat system with dummy layer used in constant force pulling simulations. Dummy atoms are shown in purple, phosphate beads are shown in red, and all other lipid beads are represented in cyan.

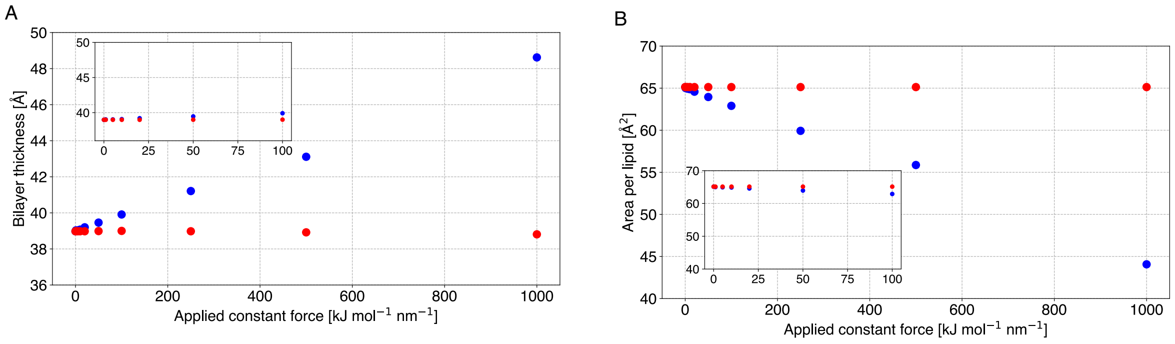

Figure S3: Bilayer thickness (left) and area per lipid (right) estimated from the NPT-restrain (blue) and NVT-freeze (red) dummy atoms methods.

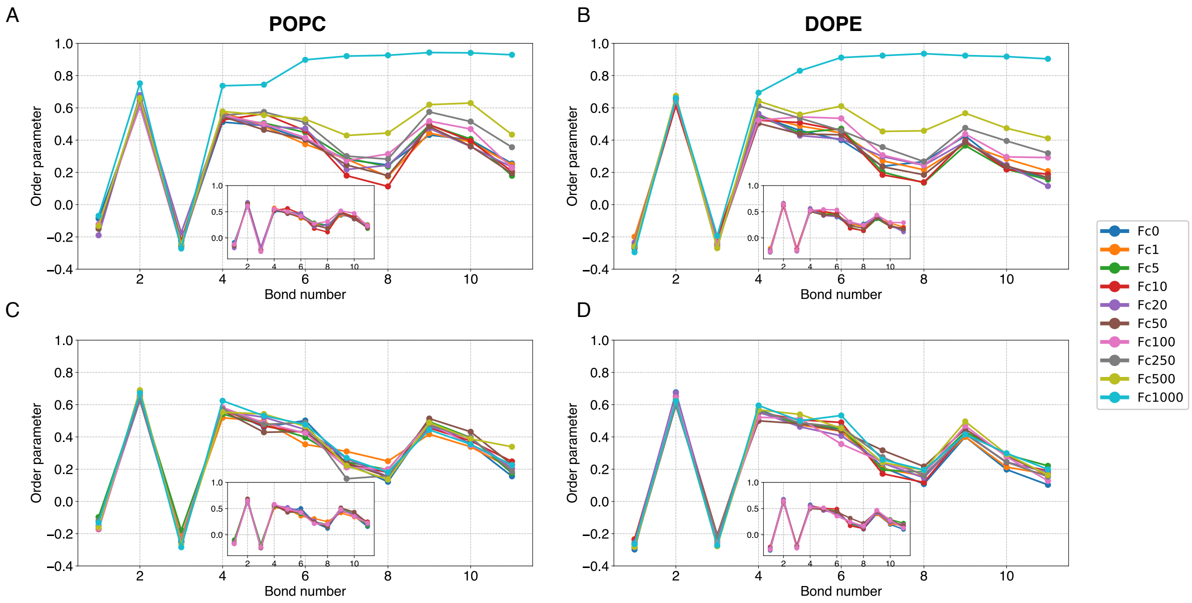

Figure S4: Lipid order parameter for POPC and DOPE lipids. The top and bottom rows show the results for the NPT-restrain and NVT-freeze dummy atoms methods, respectively.

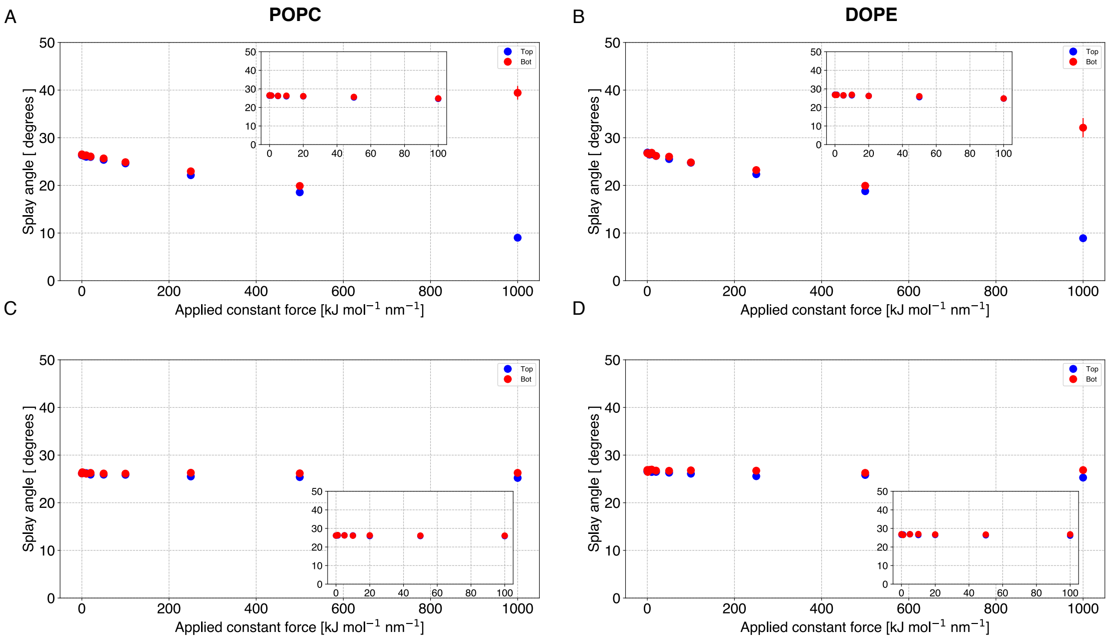

Figure S5: The lipid splay angle for POPC and DOPE for the NPT-restain method are shown in (A) and (B), respectively. The lipid splay angle for POPC and DOPE for the NVT-freeze method are shown in (C) and (D), respectively. In each subplot the splay angles for lipids in the top leaflet (blue) and bottom leaflet (red) are shown.

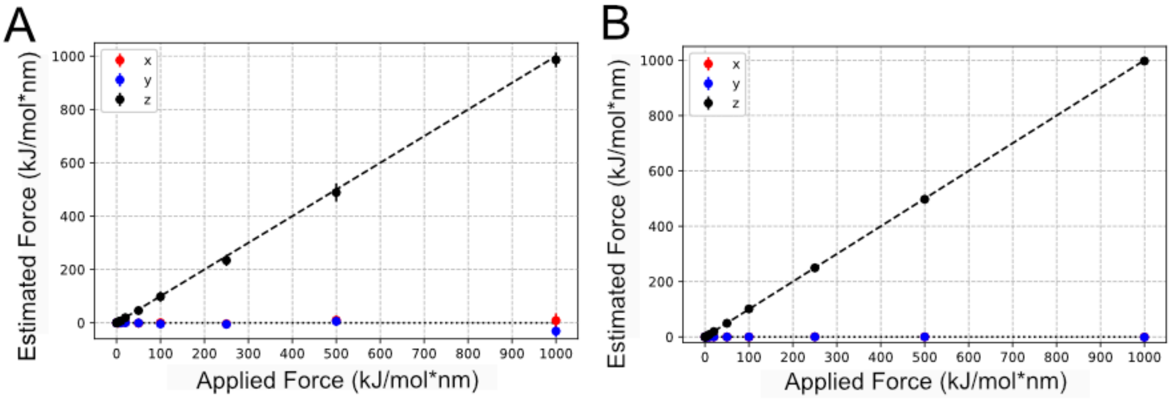

Figure S6: Dummy particle force sensing validation. (A) Estimated forces for the NPT dummy particles simulations, where the dummy particles were restrained in x, y, and z dimensions. (B) NVT dummy particles simulations, where dummy particles were frozen in x, y, and z directions.

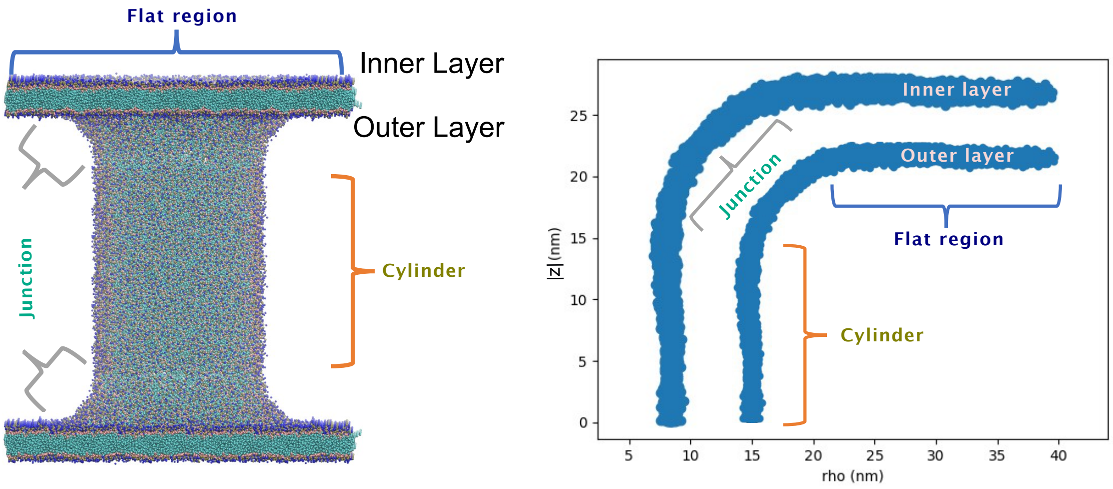

Figure S7: IMM model and coordinate transformation from three dimensional coordinates (left) to 2D polar coordinates (right).

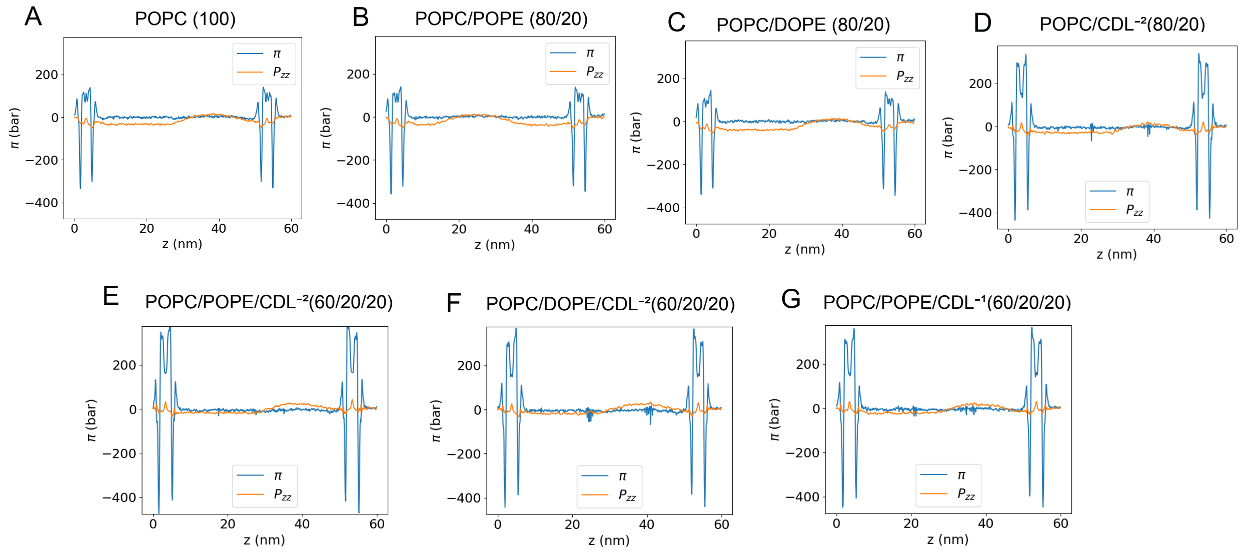

Figure S8: Lateral pressure profiles π and the z-component of the pressure tensor (P_{zz}) as a function of z-direction. Values for P_{zz} are shifted by computing $P_{zz}(z) - P_{zz}(L_z)$, where L_z is the top of the box. Pressures were computed and averaged over the final 1 μs of the simulations of IMM with r_{cyl} = 10nm

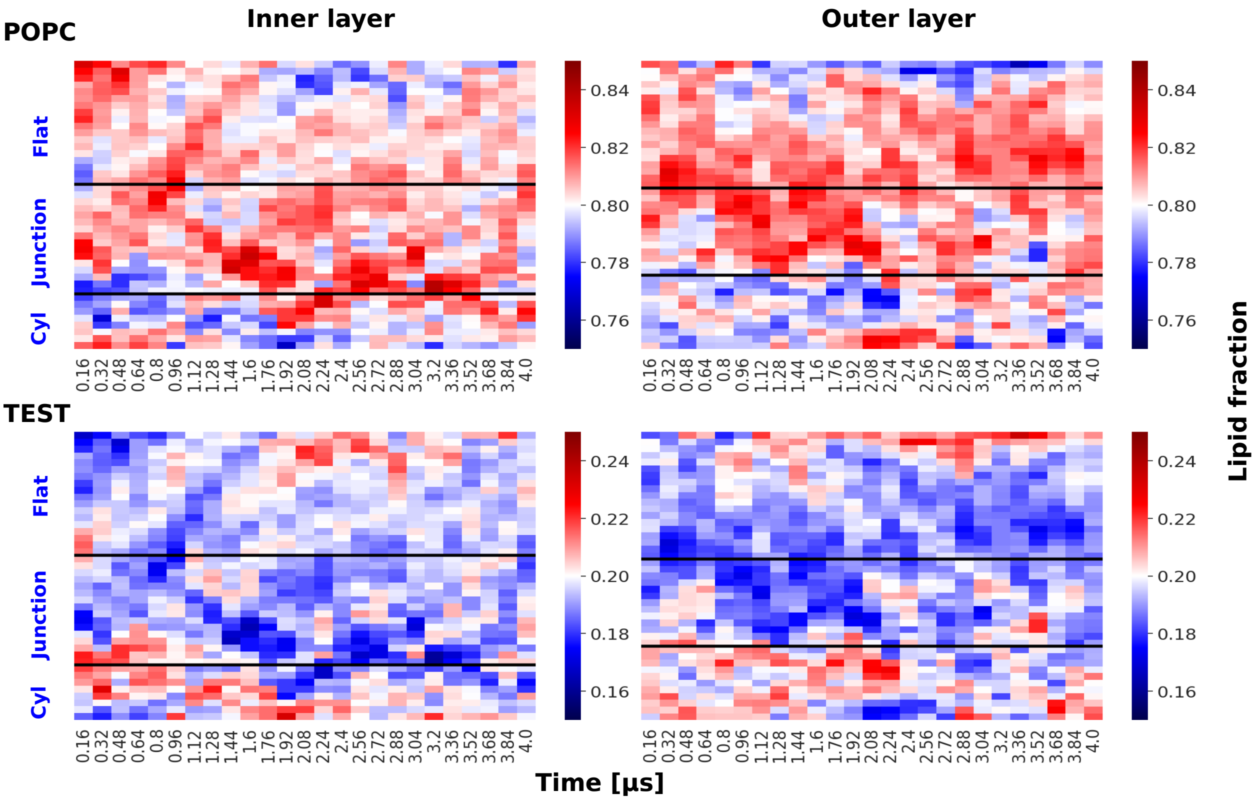

Figure S9: Dynamics of lipid partitioning of POPC and Test (POPC) lipids in a POPC/Test (4:1) IMM system over the 4 μs simulation time.

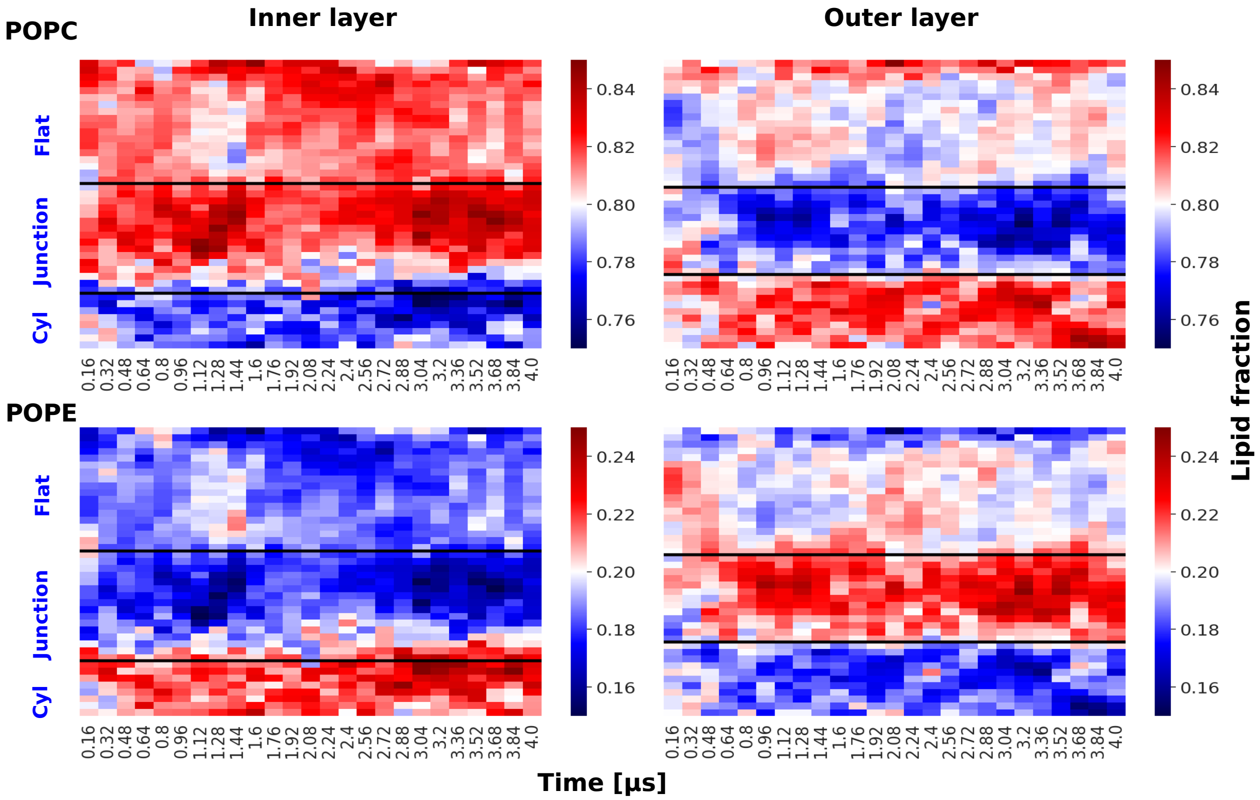

Figure S10: Dynamics of lipid partitioning of POPC and POPE lipids in a POPC/POPE (4:1) IMM system over the 4 μs simulation time.

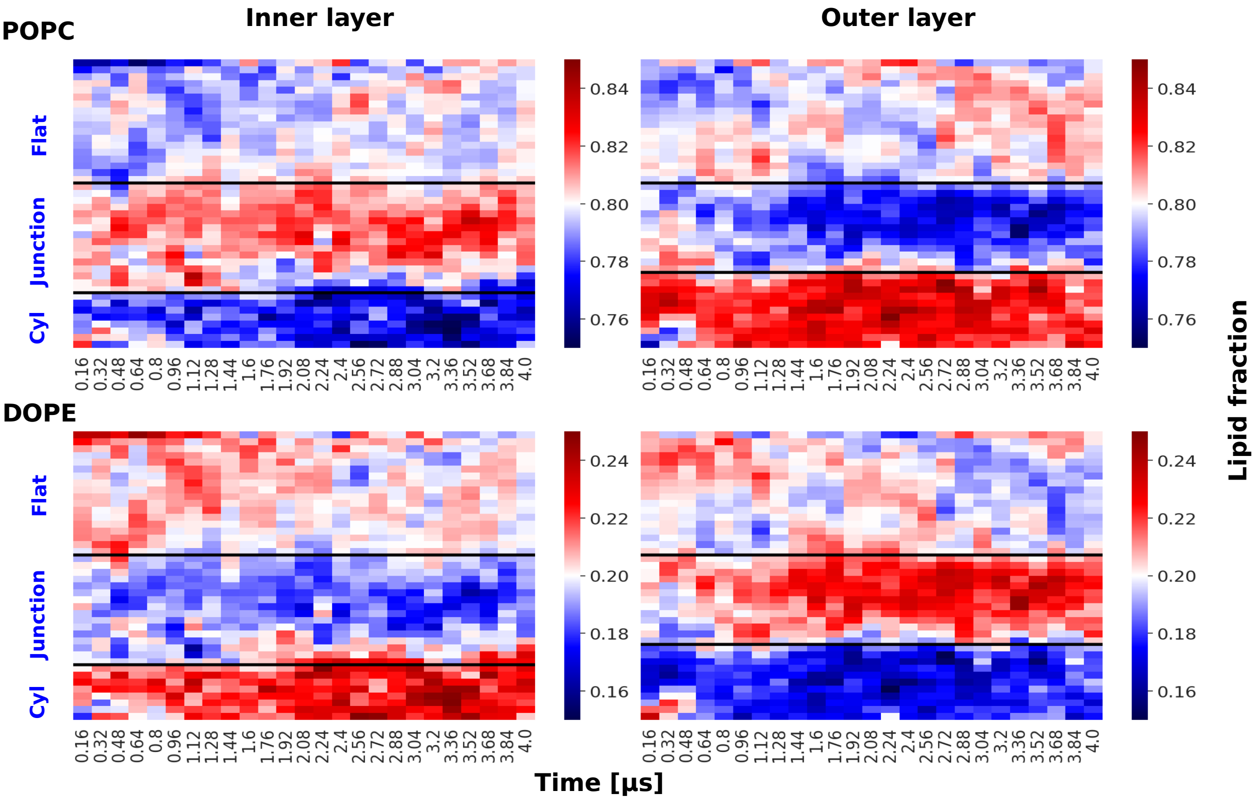

Figure S11: Dynamics of lipid partitioning of POPC and DOPE lipids in a POPC/DOPE (4:1) IMM system over the 4 μs simulation time.

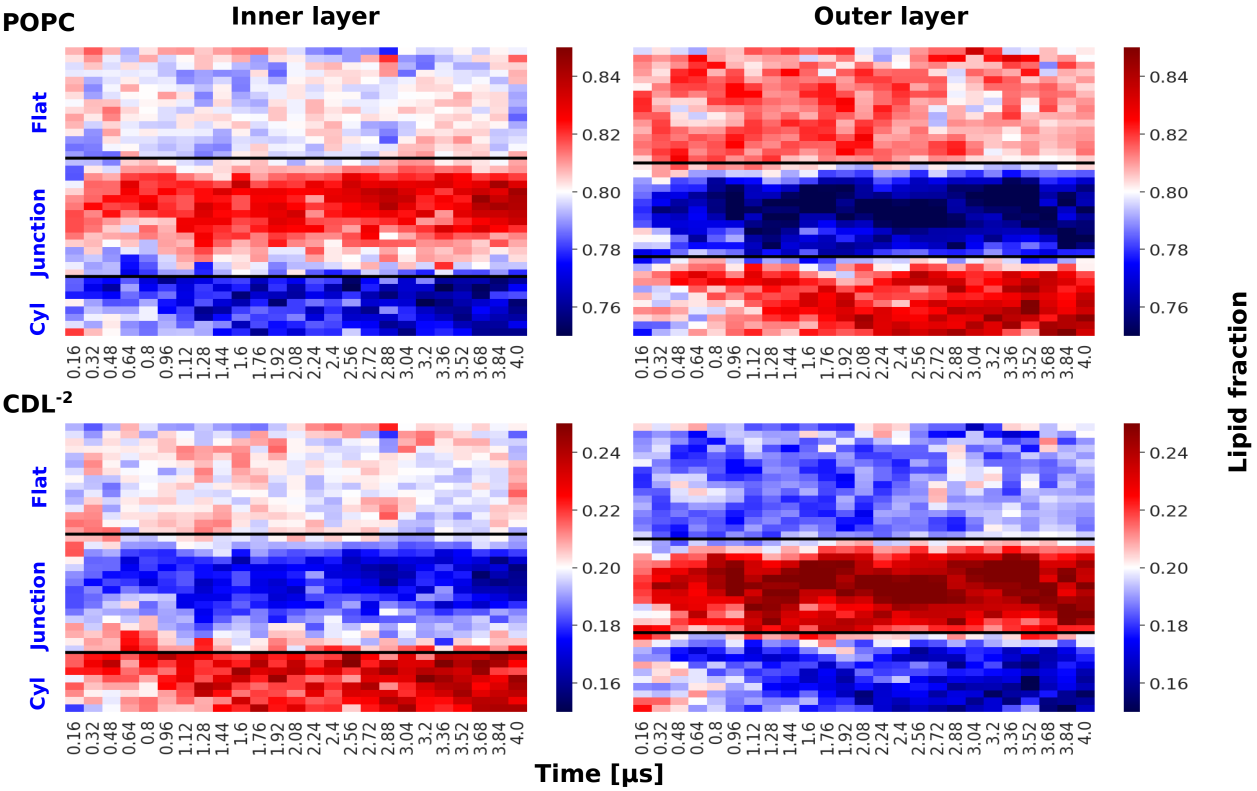

Figure S12: Dynamics of lipid partitioning of POPC and CDL^{-2} lipids in a POPC/CDL^{-2} (4:1) IMM system over the 4 μs simulation time.

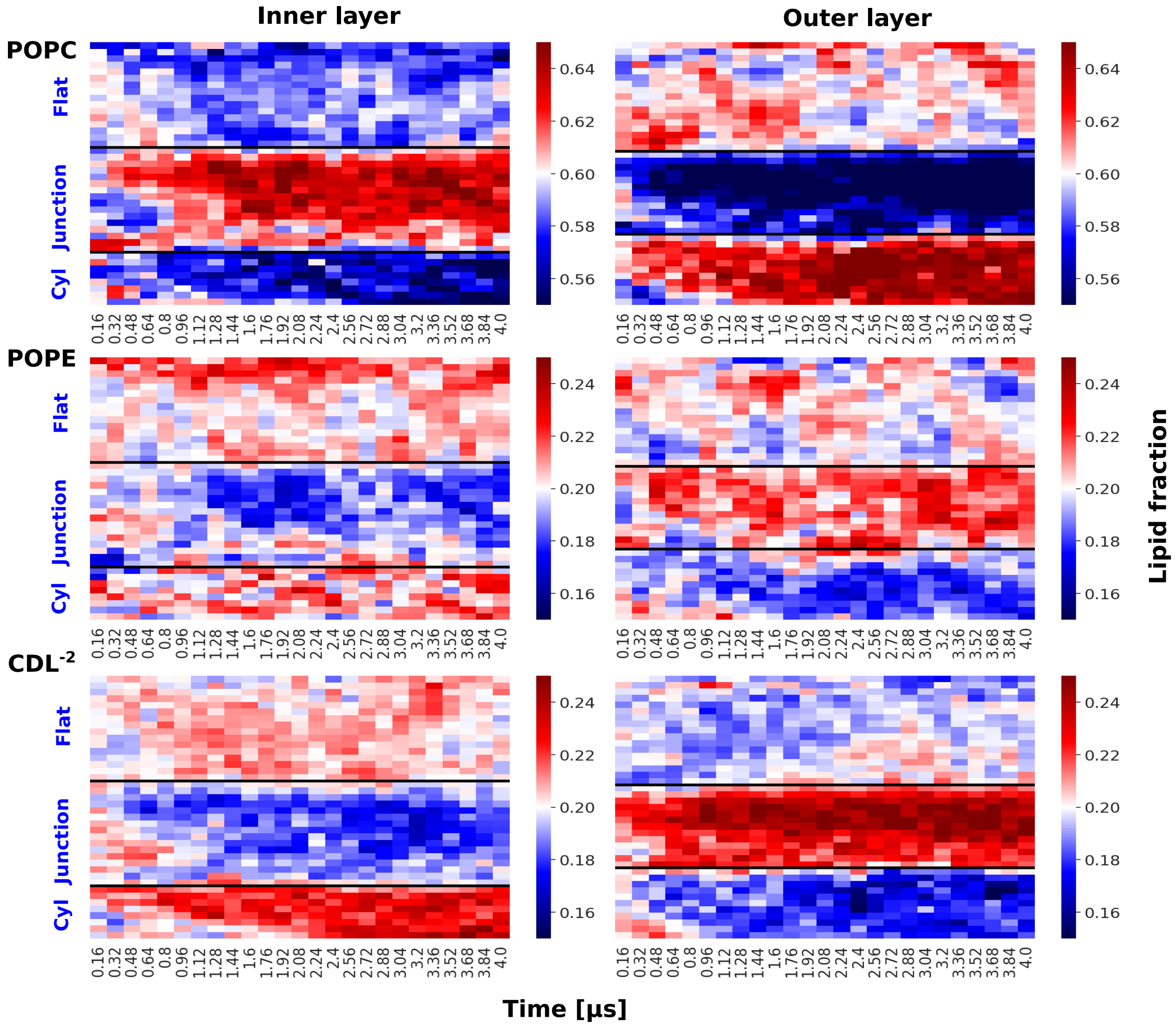

Figure S13: Dynamics of lipid partitioning of POPC, POPE, and CDL^{-2} lipids in a POPC/POPE/CDL^{-2} (3:1:1) IMM system over the 4 μs simulation time.

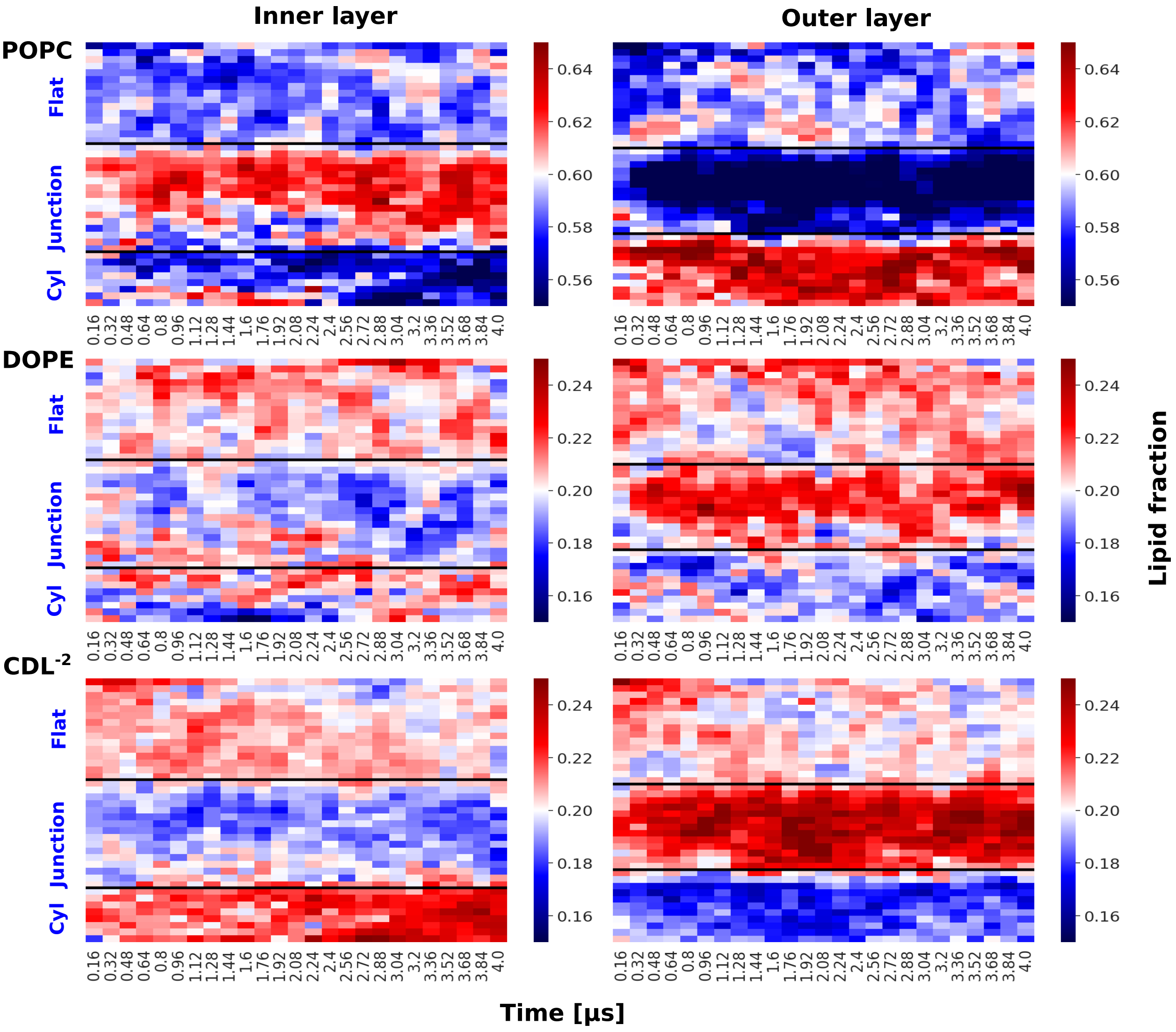

Figure S14: Dynamics of lipid partitioning of POPC, DOPE, and CDL^{-2} lipids in a POPC/DOPE/CDL^{-2} (3:1:1) IMM system over the 4 μs simulation time.

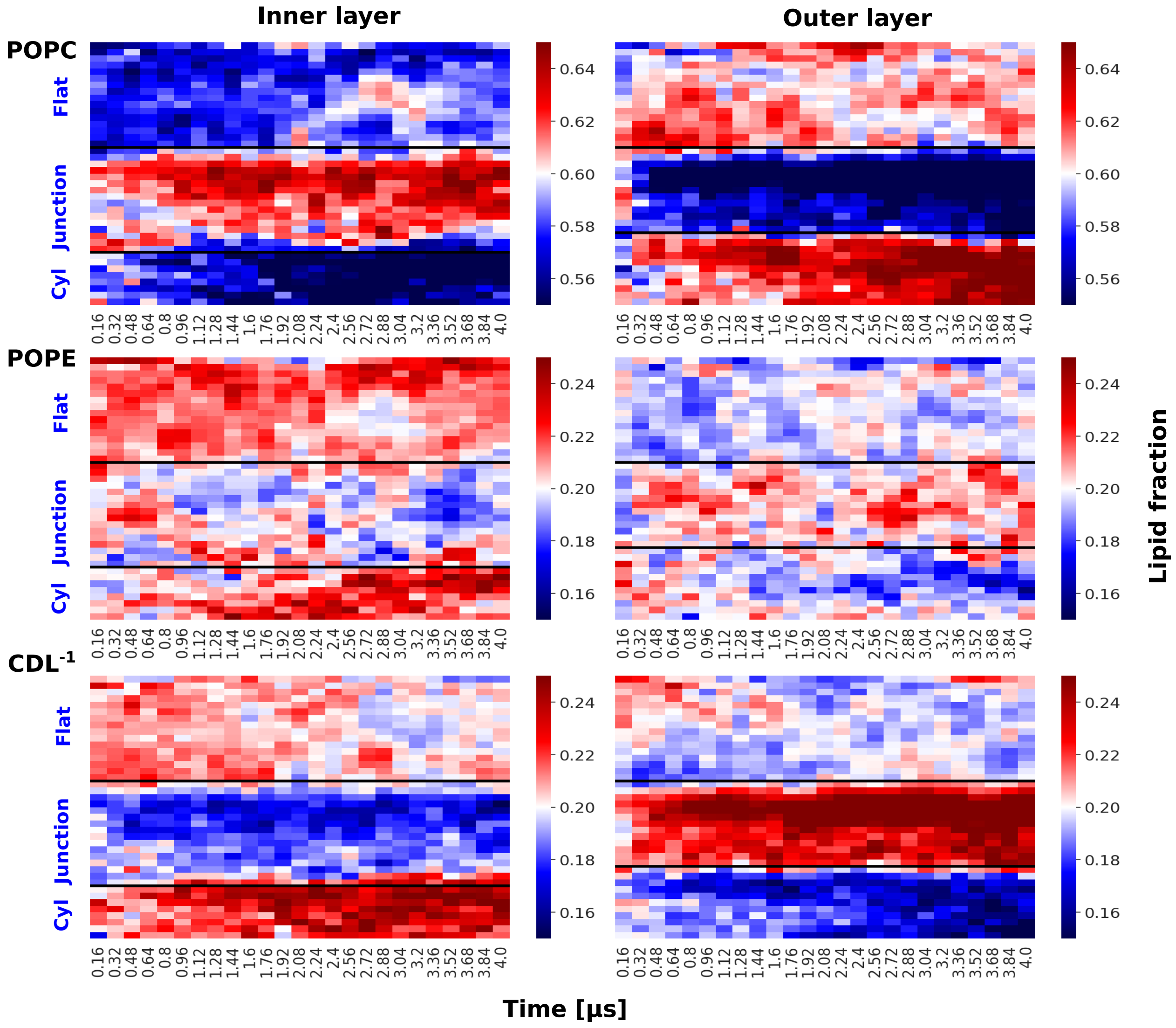

Figure S15: Dynamics of lipid partitioning of POPC, POPE, and CDL^{-1} lipids in a POPC/POPE/CDL^{-1} (3:1:1) IMM system over the 4 μs simulation time.

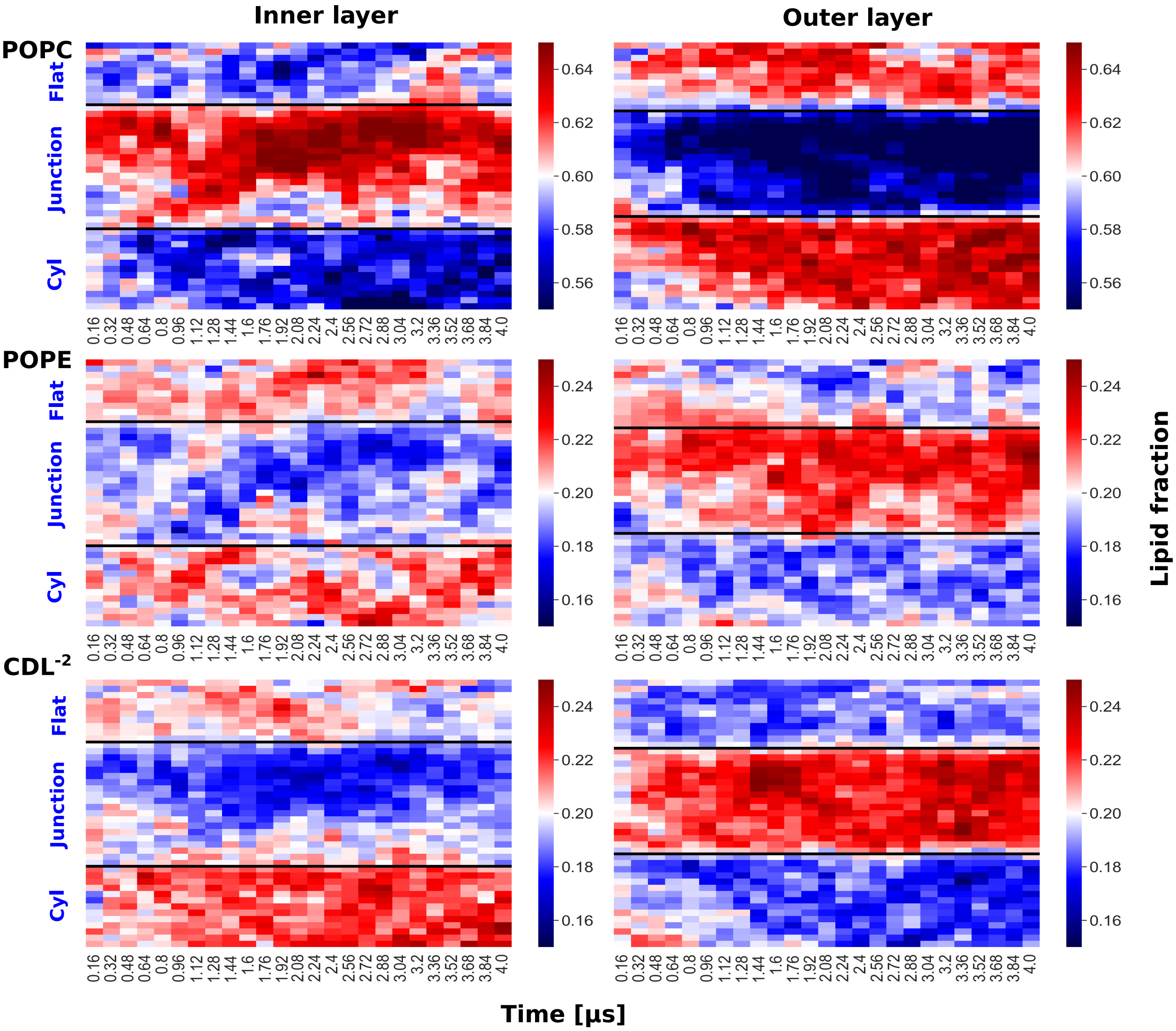

Figure S16: Dynamics of lipid partitioning of POPC, POPE, and CDL^{-2} lipids in a POPC/POPE/CDL^{-2} (3:1:1) IMM system, with a 15 nm cylinder radius, over the 4 μs simulation time.

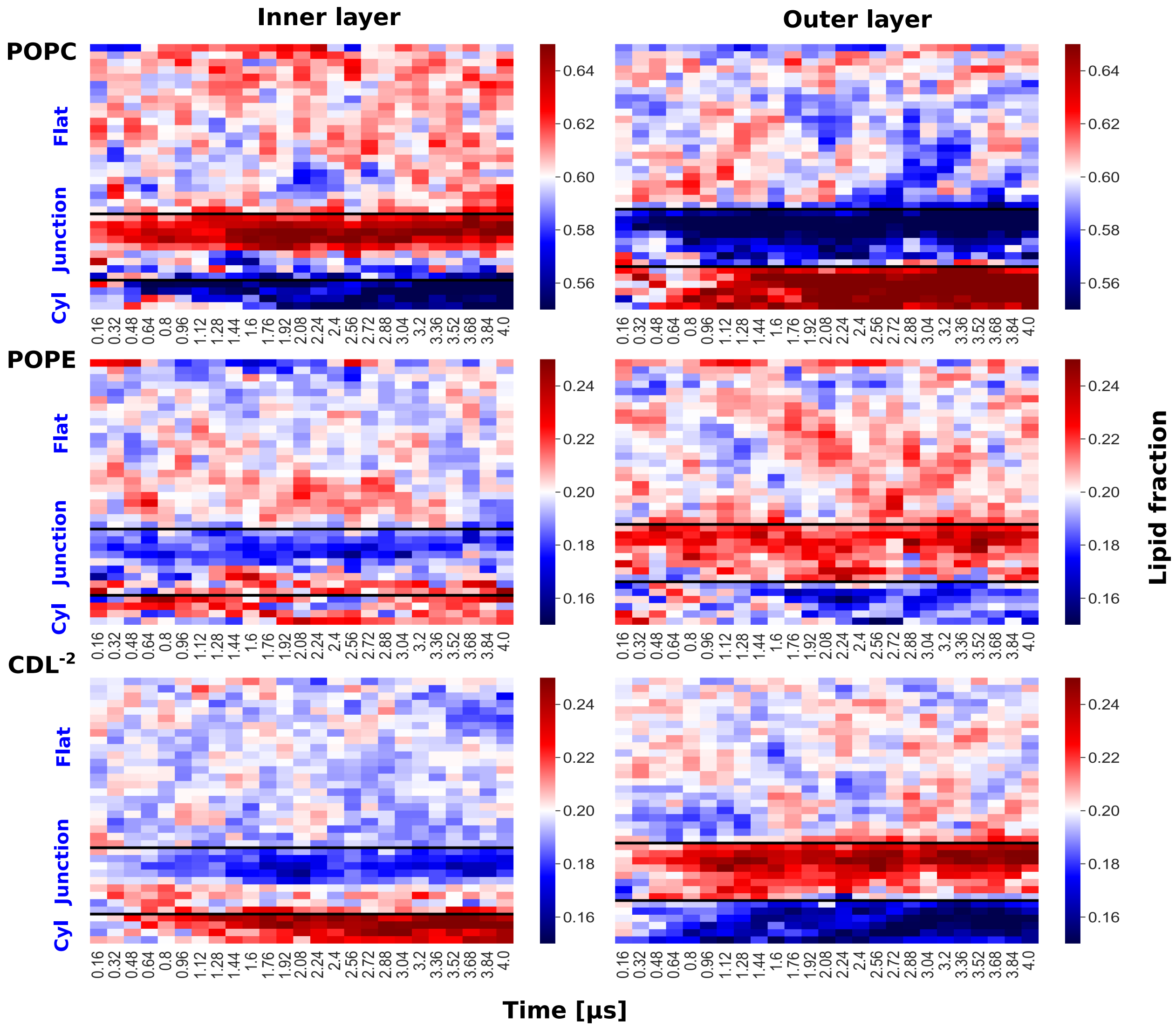

Figure S17: Dynamics of lipid partitioning of POPC, POPE, and CDL^{-2} lipids in a POPC/POPE/CDL^{-2} (3:1:1) IMM system, with a 5 nm cylinder radius, over the 4 μs simulation time.

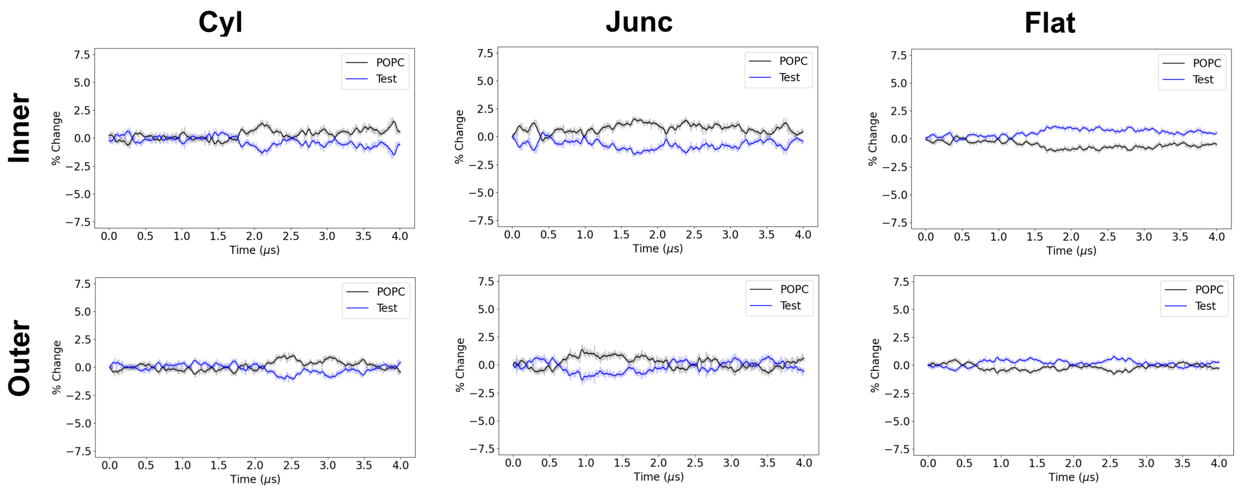

Figure S18: Compartmental analysis for POPC/Test (4:1) IMM system. The percentage change for all lipids in all compartments of both inner and outer leaflets is calculated over the 4 μs trajectory. The solid lines are running averages computed using a 40 ns window, and the transparent line is the raw data.

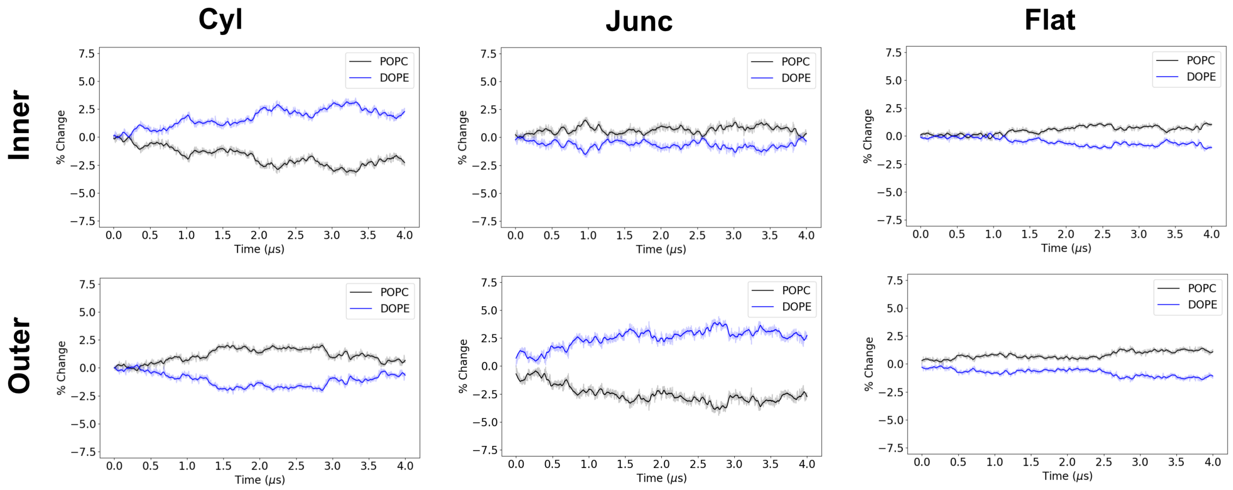

Figure S19: Compartmental analysis for POPC/DOPE (4:1) IMM system. The percentage change for all lipids in all compartments of both inner and outer leaflets is calculated over the 4 μs trajectory. The solid lines are running averages computed using a 40 ns window, and the transparent line is the raw data.

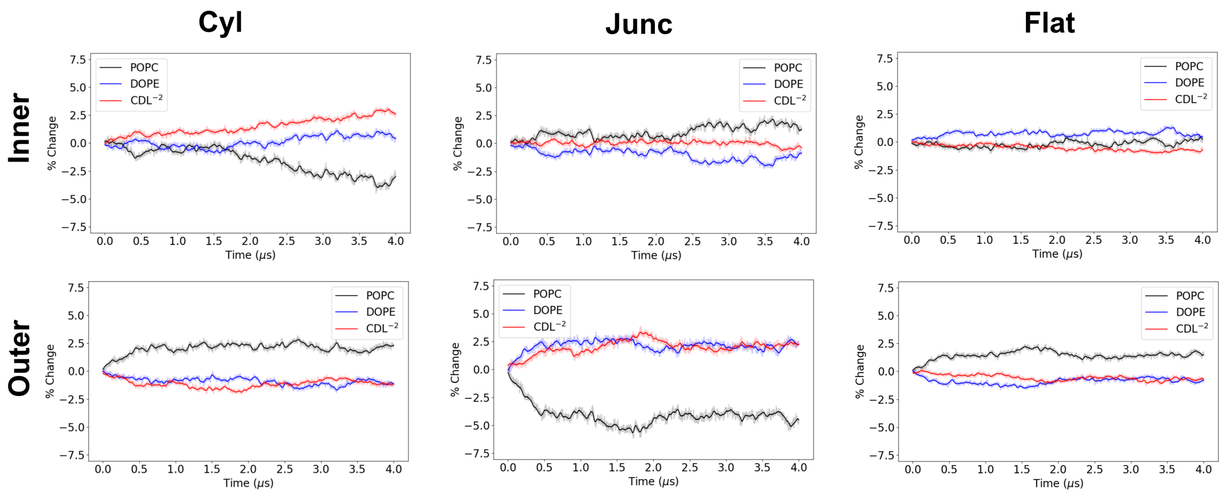

Figure S20: Compartmental analysis for POPC/DOPE/CDL^{-2} (3:1:1) IMM system. The percentage change for all lipids in all compartments of both inner and outer leaflets is calculated over the 4 μs trajectory. The solid lines are running averages computed using a 40 ns window, and the transparent line is the raw data.

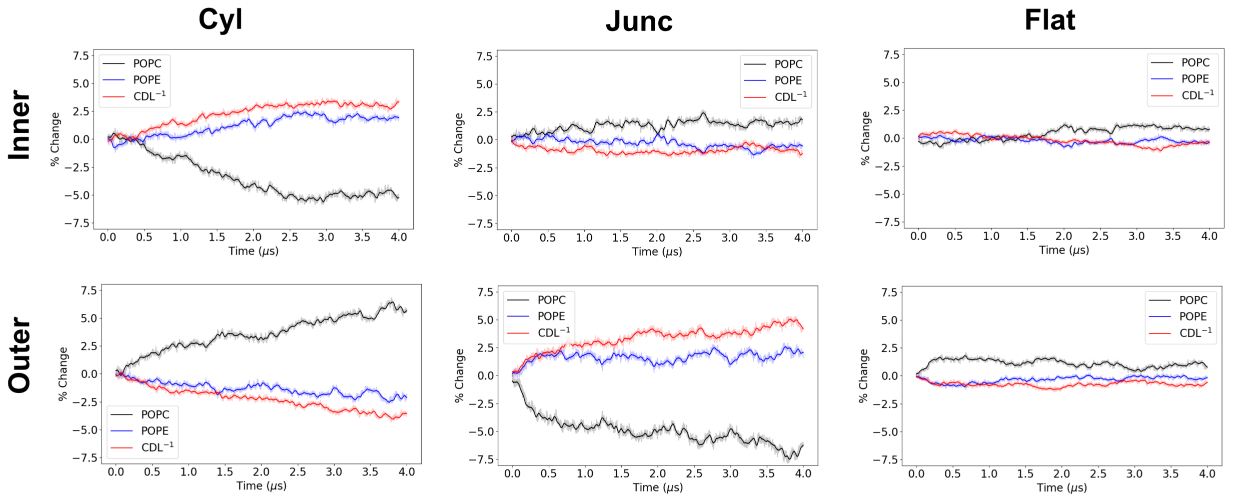

Figure S21: Compartmental analysis for POPC/POPE/CDL^{-1} (3:1:1) IMM system. The percentage change for all lipids in all compartments of both inner and outer leaflets is calculated over the 4 μs trajectory. The solid lines are running averages computed using a 40 ns window, and the transparent line is the raw data.

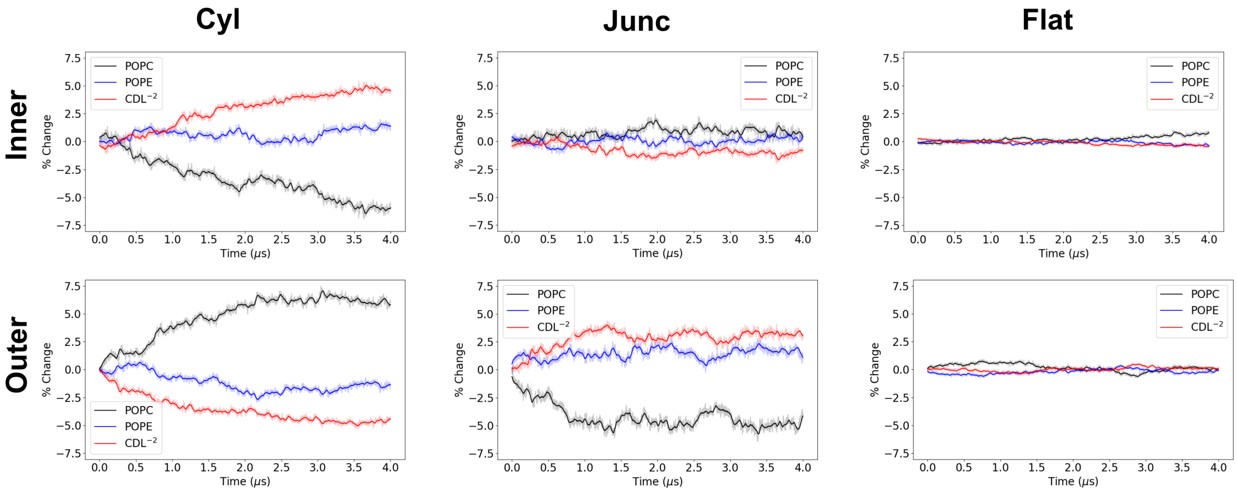

Figure S22: Compartmental analysis for POPC/POPE/CDL^{-2} (3:1:1) IMM system with 5 nm cylinder radius. The percentage change for all lipids in all compartments of both inner and outer leaflets is calculated over the 4 μs trajectory. The solid lines are running averages computed using a 40 ns window, and the transparent line is the raw data.

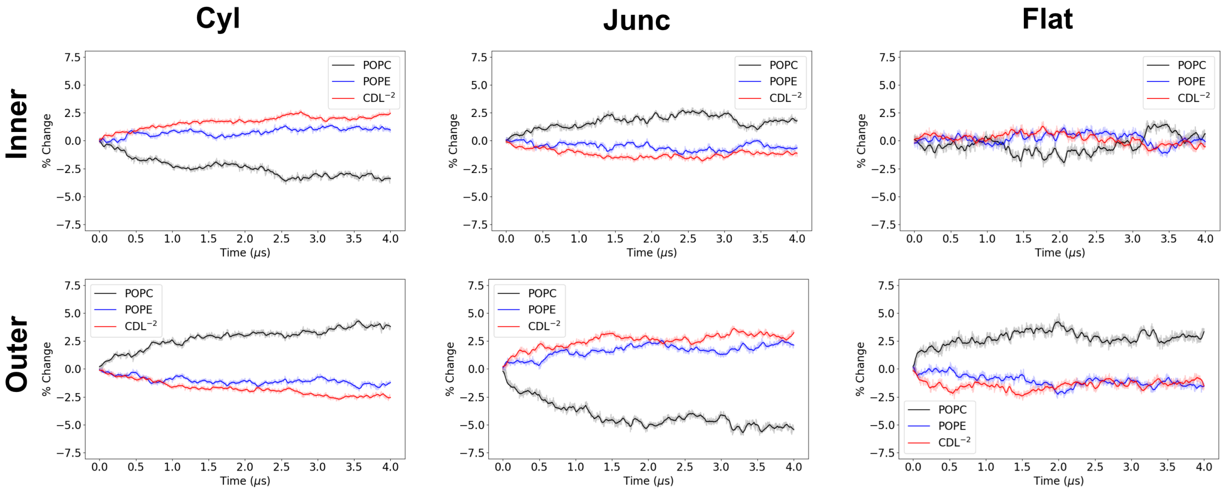

Figure S23: Compartmental analysis for POPC/POPE/CDL^{-2} (3:1:1) IMM system with 15 nm cylinder radius. The percentage change for all lipids in all compartments of both inner and outer leaflets is calculated over the 4 μs trajectory. The solid lines are running averages computed using a 40 ns window, and the transparent line is the raw data.

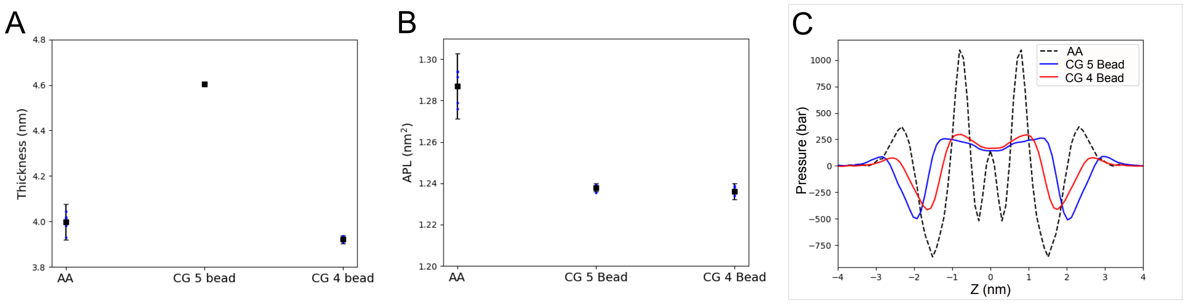

Figure S24: Validation of CDL 4-bead acyl chain model. The area per lipid (APL), bilayer thickness, and lateral pressure profiles (LPP) are shown in A, B, and C, respectively. These data are extracted from simulations of bilayers of pure CDL^{-2} lipids. The all-atom (AA) simulations were performed using the CHARMM36 forcefield and were previously described in ref. 20. The AA system contains 100 lipids, while the CG systems contain 200 lipids. APL and thickness analyses were performed over the final 100 ns of data, and errors were computed as 95% confidence intervals based on block averaging using 20 ns blocks. The mean values are shown as black squares and the individual blocks as smaller blue circles. The 4-bead model shows good agreement with the AA thickness, and the location of peaks in the 4-bead LPP is better aligned with the AA data.
