# Supplementary material for: Curvature sensing lipid dynamics in a mitochondrial inner membrane model
Source: Commun Biol. 2024 Jan 5;7:29. doi: 10.1038/s42003-023-05657-6 (PMC10770132; doi:10.1038/s42003-023-05657-6)
Supplement: Supplementary file 3 — Description of Additional Supplementary Files [file 42003_2023_5657_MOESM3_ESM.pdf]

Description of Additional Supplementary Files

File name: Supplementary Data 1
Description: MARTINI .itp file for CDL-2 with 4 tail beads per acyl chain.

File name: Supplementary Data 2
Description: MARTINI .itp file for CDL-1 with 4 tail beads per acyl chain.
